# Comparison of urinary TIMP-2 and IGFBP7 cut-offs to predict acute kidney injury in critically ill patients: A PRISMA-compliant systematic review and meta-analysis

**DOI:** 10.1097/MD.0000000000016232

**Published:** 2019-06-28

**Authors:** Dongquan Zhang, Yuan Yuan, Longfei Guo, Quanhong Wang

**Affiliations:** Intensive Care Unit, Gansu Provincial Hospital, Lanzhou, Gansu Province, PR China.

**Keywords:** acute kidney injury, cut-off, diagnosis, IGFBP7, TIMP-2

## Abstract

**Background:**

Tissue inhibitor of metalloproteinase-2 (TIMP-2) and insulin-like growth factor binding protein 7 (IGFBP7) are recently identified urinary biomarkers of acute kidney injury (AKI) in critically ill patients. Because their predictive accuracies vary widely, a meta-analysis was performed to evaluate the accuracy of previously reported urinary TIMP-2 and IGFBP7 cut-offs for predicting AKI.

**Methods:**

This meta-analysis was reported following the guideline of PRISMA. Four databases, PubMed, the Cochrane Library, the ISI Web of Knowledge, and Embase, were systematically searched from inception to June 2018 by 2 investigators, who independently selected studies, extracted relevant data, and evaluated study quality. A bivariate model was used to calculate the pooled estimates.

**Results:**

The search identified 5 studies with 1619 critically ill patients. Urinary TIMP-2 and IGFBP7 cut-off points of 0.3 (ng/ml)^2^/1000 had a sensitivity of 0.89 [95% confidence interval (CI) 0.85–0.93], a specificity of 0.48 (95% CI 0.45–0.51) and a diagnostic odds ratio (DOR) of 8.33 (95% CI 5.55–12.52). The area under the curve (AUC) estimated by the summary receiver operating characteristic (SROC) curve was 0.748. Based on 891 critically ill patients from 4 studies, urinary TIMP-2 and IGFBP7 cut-off points of 2.0 (ng/ml)^2^/1000 had a sensitivity of 0.45 (95% CI 0.37–0.53), a specificity of 0.93 (95% CI 0.91–0.95) and a DOR of 11.43 (95% CI 7.43–17.57). The AUC estimated by SROC was 0.844.

**Conclusion:**

Cut-off values around 0.3 (ng/ml)^2^/1000 (high sensitivity) and 2.0 (ng/ml)^2^/1000 (high specificity) could be accurate surrogate biomarkers predicting AKI in critically ill patients. The urinary TIMP-2 and IGFBP7 cut-off point of 2.0 (ng/mL)^2^/1000 appears to have the highest overall accuracy.

**Trial registration:**

PROSPERO registration number 2018: CRD42018084457 Registered on 11 February 2018.

## 1 Introduction

Acute kidney injury (AKI) is a common disorder of critically ill patients, especially those in the intensive care unit (ICU), and is a potentially life-threatening factor closely associated with prolonged ICU stay, severe complications, and increased mortality.^[1]^ The prevention of AKI and its diagnosis in early stages are essential to improve the prognosis of critically ill patients. Although considered standard tools in routine clinical tests, serum creatinine concentration and urine output are not suitable for the early detection of AKI, owing to inherent methodological problems.^[2]^ Many potential biomarkers for the early detection of AKI have been identified,^[3]^ including neutrophil gelatinase-associated lipocalin (NGA L),^[4]^ N-acetyl-b-D-glucosaminidase (NAG),^[5]^ interleukin 18 (IL-18),^[6]^ glutathione S-transferase (GST),^[7]^ kidney injury molecule-1 (KIM-1),^[8]^ and liver-type fatty acid-binding protein (L-FABP).^[9]^ More recently, urinary tissue inhibitor of metalloproteinase-2 (TIMP-2) and insulin-like growth factor binding protein 7 (IGFBP7) were reported to be superior to other biomarkers of AKI.^[10]^

The novel AKI-related biomarkers TIMP-2 and IGFBP7, which have been reported to induce G1 cell cycle arrest, can indicate a pre-injury status that could lead to AKI. A corresponding commercially available test kit has been approved by the US Food and Drug Administration (FDA).^[11]^ TIMP-2 and IGFBP7 are both secreted under conditions of cellular stress during the early stages of tubular cell injury caused by various insults, including inflammation, ischemia, oxidative stress, drugs, and toxins.^[12]^ These findings suggest that urinary TIMP-2 and IGFBP7 levels may have potential value in the early diagnosis of AKI.

Several studies have reported that the concentrations of TIMP-2 and IGFBP7 proteins in urine are early indicators of AKI in critically ill patients.^[13–17]^ However, these studies differed markedly, including in their urinary TIMP-2 and IGFBP7 threshold concentrations, making the urinary TIMP-2 and IGFBP7 levels more reliably associated with AKI unclear. This systematic review and meta-analysis therefore evaluated the accuracy of urinary TIMP-2 and IGFBP7 in diagnosing AKI in critically ill patients.

## 2 Methods

The present meta-analysis was conducted and reported according to the guidelines of the Meta-analysis of Observational Studies in Epidemiology and the Preferred Reporting Items for a Systematic Review and Meta-analysis of Diagnostic Test Accuracy Studies (PRISMA-DTA).^[18]^ The review protocol was registered by the PROSPERO registry of systematic reviews (registry number 2018: CRD42018084457). Ethical approval was not necessary because this study was a meta-analysis. Therefore, our data were based on published studies only.

### 2.1 Data sources

The PubMed, Cochrane Library, ISI Web of Knowledge and Embase databases were systematically searched from inception to June 2018. The search strategy used the keywords (“TIMP-2” OR “tissue inhibitor metalloproteinase-2” OR “IGFBP7” OR “IGF-binding protein 7” OR “insulin-like growth factor binding protein 7” OR “cycle arrest biomarkers”) AND (“acute kidney injury” OR “AKI” OR “acute renal failure” OR “ARF” OR “acute kidney disease” OR “acute kidney stress”) AND (intensive care OR ICU OR critically ill OR intensive care unit OR critical care OR severely ill). All terms were searched as keywords and MeSH headings where available. References of included studies and relevant reviews were manually searched for additional suitable publications.

### 2.2 Eligibility criteria

Two reviewers independently evaluated studies to determine their eligibility for inclusion in the meta-analysis. In cases of disagreement, a consensus was reached by discussion or by consultation with a third reviewer. Trials were included if they:

1. reported concentrations of TIMP-2 and IGFBP7 diagnostic for AKI in adult patients (≥18 years) located in critical care setting;
2. contained sufficient information to construct a 2 × 2 contingency table (i.e., false and true positives and false and true negatives); and
3. AKI was diagnosed by the criteria of the Kidney Disease: Improving Global Outcomes (KDIGO)^[19]^ or the Acute Kidney Injury Network (AKIN).^[20]^

All possible qualified studies were considered, regardless of language. Studies were excluded if they described irrelevant research or animal experiments. Also excluded were review articles, meeting abstracts, studies of pediatric patients, studies of AKI in the clinical setting of cardiac surgery or emergency department or emergency room, and studies with insufficient information to extract data, even after contacting the corresponding authors.

### 2.3 Data extraction

Using standard forms, 2 reviewers independently extracted study characteristics and data from each eligible study. This information included the study authors, year of the study, country of origin, study design, population setting, sample size, numbers of patients with and without AKI, mean ages, and mean baseline serum creatinine (SCr) concentrations. The investigators also recorded the AKI definition used in each study, as well as their AKI threshold and use of blinding. Also recorded were times of measuring TIMP-2 and IGFBP7 concentrations, methods of measurement, cut-off concentrations, and diagnostic accuracy estimates, including numbers of true-positives (TP), false-positives (FP), false-negatives (FN), and true-negatives (TN). Other parameters recorded were sensitivities, specificities, and AUCs for each reported test threshold.

### 2.4 Quality assessment

The quality of primary studies was assessed using the Quality Assessment of Diagnostic Accuracy Studies 2 (QUADAS-2) checklist,^[21]^ recommended by the Cochrane collaboration for the quality assessment of diagnostic studies. The QUADAS-2 tool consists of 4 domains: patient selection, index test, reference standard, and flow and timing. Each domain assesses the risk of bias by representative questions, whose corresponding answers may be “yes,” “no,” or “unclear.” If all answers were “yes” in one domain, then there was a low risk of bias. The inclusion of a “no” in any domain indicated a high risk of bias. Studies not providing sufficient information to answer “yes” or “no” in the domain were regarded as having an unclear risk of bias. These assessments were performed independently by 2 reviewers, with disagreements resolved by consensus.

### 2.5 Statistical analysis

Random effects bivariate models were utilized for separate meta-analyses of the pre-specified cut-off values. The diagnostic accuracies of indices of TIMP-2 and IGFBP7 were computed for each study. Parameters calculated included the sensitivity, specificity, positive likelihood ratio (PLR), negative likelihood ratio (NLR) and diagnostic odds ratio (DOR). A PLR>1 for a positive test result was associated with the presence of disease, and an NLR<1 for a negative test result was associated with the absence of disease. The DOR is a single indicator that summarizes the diagnostic accuracy of a test, with higher values indicating better test performance. The overall performance of TIMP-2 and IGFBP7 cut-offs was evaluated by calculating summary receiver operating characteristics curves (SROC). A Fagan nomogram was applied based on a pre-test probability of 20%. Heterogeneity among studies was evaluated by the inconsistency test (*I*^2^), with low, moderate, and high heterogeneity occurring when *I*^2^ was 0–25%, 26–50%, and 51–75%, respectively.^[22]^ Publication bias was tested using Deeks’ funnel plots. A *P* value <.05 was considered statistically significant in all analyses except for the Deeks’ test, where a *P* value <.1 was considered statistically significant.

To evaluate whether the diagnostic accuracy of urinary TIMP-2 and IGFBP7 cut-offs for predicting AKI was modified by clinical characteristics, Prior sensitivity analysis were specified based on definition of AKI (KIDGO vs AKIN), blind method (yes vs no), AKI threshold (AKI stage 2 or 3 vs AKI stage 1), time of measurement (within 12 hours vs within 24 hours).

All analyses were performed using Meta-DiSc version 1.4 (Universidad Complutense, Madrid, Spain) and Stata version. 12.1 software (Stata, College Station, TX) by metandi and midas commands.

## 3 Results

### 3.1 Identification of studies

**Figure 1:**
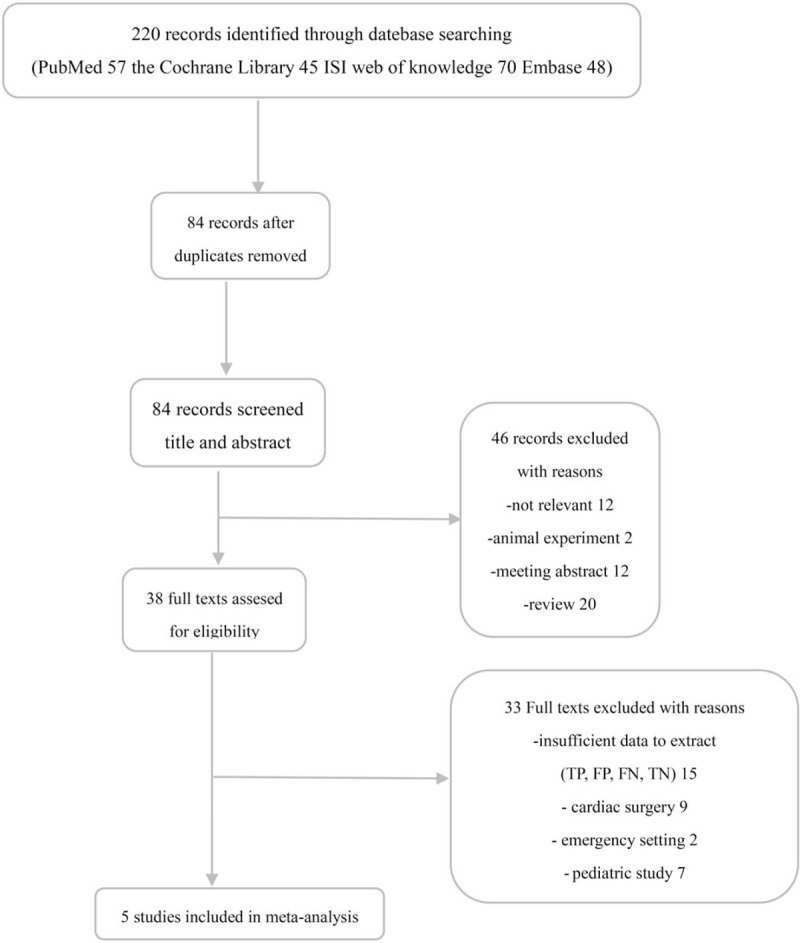
Flow diagram of literature search and selection process of the studies.

The flow chart of the study selection procedure is shown in Figure 1. An initial comprehensive search of the database and a review of abstracts identified 38 clinical studies. Fifteen articles were excluded because of insufficient data to construct a 2 × 2 contingency table, 9 were excluded because they assessed patients who underwent cardiac surgery, and 2 were excluded because they located in emergency department or emergency room, and 7 were excluded because they were studies of pediatric populations. Finally, 5 articles^[13–17]^ were included in the meta-analysis. All 5 studies were published as full-text articles in peer-reviewed journals.

### 3.2 Characteristics of the included studies

Details regarding all 5 included studies, involving a total of 1619 patients, are presented in Table 1. All 5 were prospective cohort studies published between 2013 and 2017, including 2 performed in the USA,^[14,15]^ 2 in North America and Europe,^[13,16]^ and one in Spain.^[17]^ The number of patients varied from 98 to 728 and the reported mean or median age of the included patients ranged from 55 to 65 years. Mean baseline serum creatinine levels were in the reference range in all studies. AKI was defined based on KDIGO criteria, with AKI thresholds in 4 studies being AKI stage 2 or 3; in 1 study,^[17]^ however, AKI was defined according to AKIN criteria, with AKI stage 1 criteria being the primary endpoint. In 4,^[14–17]^ patients and caregivers were blinded to treatment, whereas another study ^[13]^ did not report a blinding method.

**Table 1:**
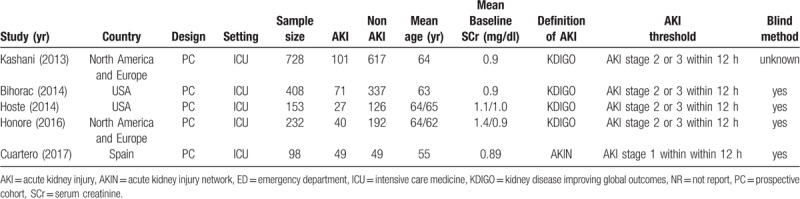
Characteristics of studies included in this meta-analysis.

The ability of urinary TIMP-2 and IGFBP7 to predict AKI was evaluated within 12 hours in 4 studies^[13–15,17]^ and within 24 hours in 1 studies.^[16]^ All 5 studies used the commercially available NephroCheck test to measure TIMP-2 and IGFBP7 concentrations in urine samples. Cut-off values for urinary TIMP-2 and IGFBP7 varied, with the most widely used cut-off values being 0.3 and 2.0 (ng/mL)^2^/1000. Time of measurement, assay method, cut-off point and diagnostic accuracy of urinary TIMP-2 and IGFBP7 in each individual study for diagnosing AKI, including TP, TN, FP and FN, sensitivity, specificity and AUC values, are listed in Table 2.

**Table 2:**
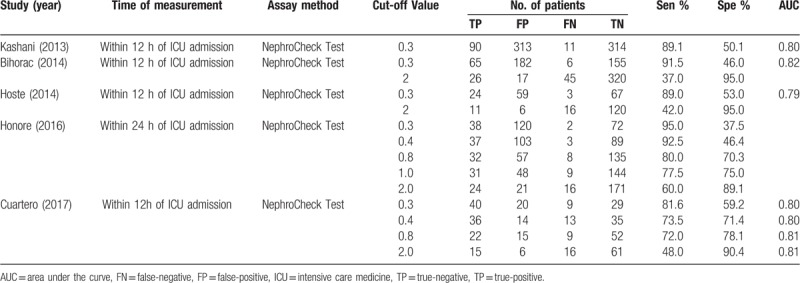
Diagnostic value of urinary TIMP-2 and IGFBP7 for acute kidney injury in individual studies.

### 3.3 Results of quality assessment

**Figure 2:**
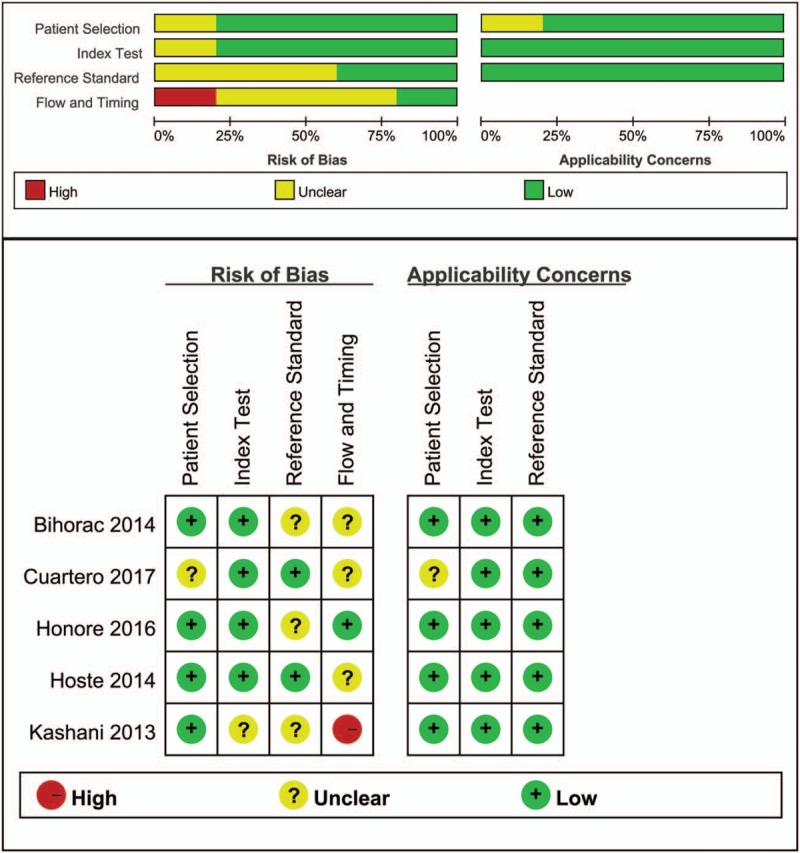
Quality assessment summary in each domain for individual studies. The quality assessment focusing on risk of bias and applicability concerns consists of 4 domains, including “patient selection,” “indextest,” “reference standard,” and “flow and timing.” Green, yellow, and red indicate low, moderate, and high risk of bias, respectively.

Figure 2 shows assessments of the methodological qualities of the included studies. Quality in all 5 studies was evaluated using the QUADAS-2 checklist. All eligible studies showed acceptable quality. One study had a high risk of bias in flow and timing.

### 3.4 Diagnostic accuracy of urinary TIMP-2 and IGFBP7 at a cut-off of 0.3 (ng/mL)^2^/1000

**Figure 3:**
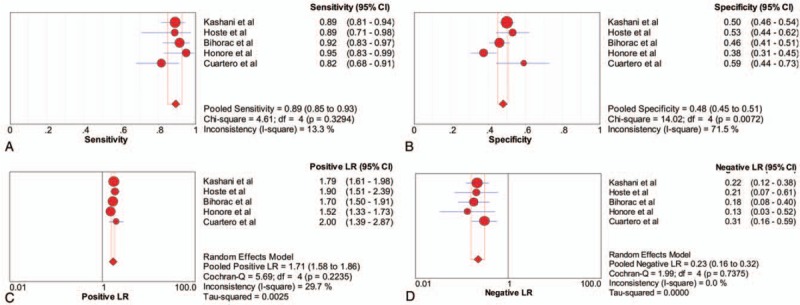
Forest plot of the sensitivity, specificity, positive likelihood ratio (PLR) and negative likelihood ratio (NLR) of urinary TIMP-2 and IGFBP7 at a cut-off of 0.3 (ng/mL)^2^/1000 for the diagnosis of acute kidney injury. (A) sensitivity, (B) specificity, (C) PLR and (D) NLR. IGFBP7 = insulin-like growth factor binding protein 7, NLR = negative likelihood ratio, PLR = positive likelihood ratio, TIMP-2 = tissue inhibitor of metalloproteinase 2.

The diagnostic accuracy for AKI of a urinary TIMP-2 and IGFBP7 cut-off of 0.3 (ng/mL)^2^/1000 was assessed in the 1619 critically ill patients included in the 5 studies. Using this cut-off, urinary TIMP-2 and IGFBP7 had a sensitivity of 0.89 (95% CI 0.85–0.93), a specificity of 0.48 (95% CI 0.45–0.51), a PLR of 1.71 (95% CI 1.58–1.86) and an NLR of 0.23 (95% CI 0.16–0.32) (Fig. 3), as well as a diagnostic odds ratio (DOR) of 8.33 (95% CI 5.55–12.52) (Fig. 5A). The AUC estimated by SROC was 0.748 (Fig. 5C). Using the DOR to explore heterogeneity caused by non-threshold effects, the Higgins heterogeneity statistic test showed low heterogeneity among the 7 studies (*I*^2^ = 0).

When the pre-test probability of 20% was combined with the PLR and NLR, the post-test probabilities of urinary TIMP-2 and IGFBP7 ≥0.3 (ng/mL)^2^/1000 and <0.3 (ng/mL)^2^/1000 for AKI were 30% and 5%, respectively (Fig. 6A). Deeks’ test (Fig. 7A) showed a statistically non-significant value (*P* = .96), indicating that there was no potential publication bias.

### 3.5 Diagnostic accuracy of urinary TIMP-2 and IGFBP7 at a cut-off of 2.0 (ng/mL)^2^/1000

**Figure 4:**
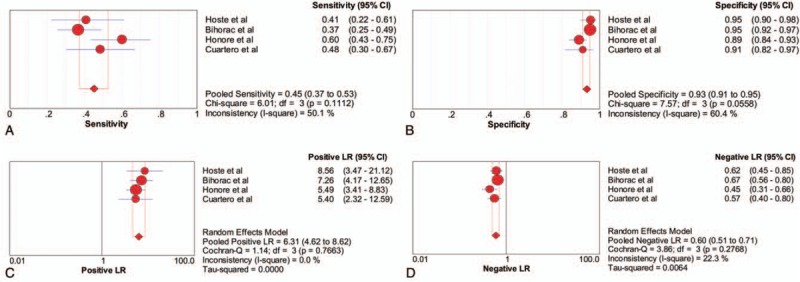
Forest plot of the sensitivity, specificity, positive likelihood ratio (PLR) and negative likelihood ratio (NLR) of urinary TIMP-2 and IGFBP7 at a cut-off of 2.0 (ng/mL)^2^/1000 for the diagnosis of acute kidney injury. (A) sensitivity, (B) specificity, (C) PLR and (D) NLR. IGFBP7 = insulin-like growth factor binding protein 7, NLR = negative likelihood ratio, PLR = positive likelihood ratio, TIMP-2 = tissue inhibitor of metalloproteinase 2.

**Figure 5:**
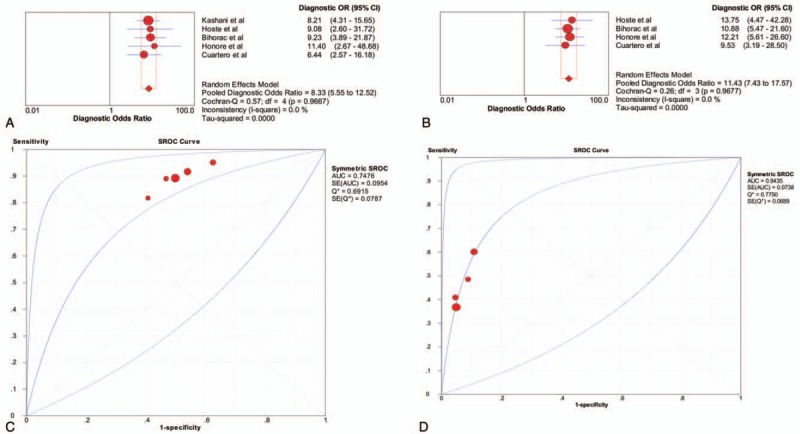
Pooled diagnostic OR (DOR) and summary receiver operating characteristic (SROC) curves of urinary TIMP-2 and IGFBP7 for acute kidney injury prediction. A comparison of pooled DOR and SROC curves between cut-off of 0.3 (ng/mL)^2^/1000 and cut-off of 2.0 (ng/mL)^2^/1000. (A) Pooled DOR in cut-off of 0.3 (ng/mL)^2^/1000. (B) Pooled DOR in cut-off of 2.0 (ng/mL)^2^/1000. (C) SROC curve of cut-off of 0.3 (ng/mL)^2^/1000. (D) SROC curve of cut-off of 0.3 (ng/mL)^2^/1000. DOR = diagnostic odds ratio, IGFBP7 = insulin-like growth factor binding protein 7, SROC = summary receiver operating characteristic, TIMP-2 = tissue inhibitor of metalloproteinase 2.

Four studies, with a total of 891 critically ill patients, assessed the diagnostic accuracy for AKI of a urinary TIMP-2 and IGFBP7 cut-off of 2.0 (ng/mL)^2^/1000. This cut-off had sensitivity of 0.45 (95% CI 0.37–0.53), a specificity of 0.93 (95% CI 0.91–0.95), a PLR of 6.31 (95% CI 4.62–8.62) and an NLR of 0.60 (95% CI 0.51–0.71) (Fig. 4). The DOR was 11.43 (95% CI 7.43–17.57) (Fig. 5B), and the AUC estimated by SROC was 0.844 (Fig. 5D). Using the DOR to explore heterogeneity caused by non-threshold effects, the Higgins heterogeneity statistic test showed low heterogeneity among the 6 studies (*I*^2^ = 0).

**Figure 6:**
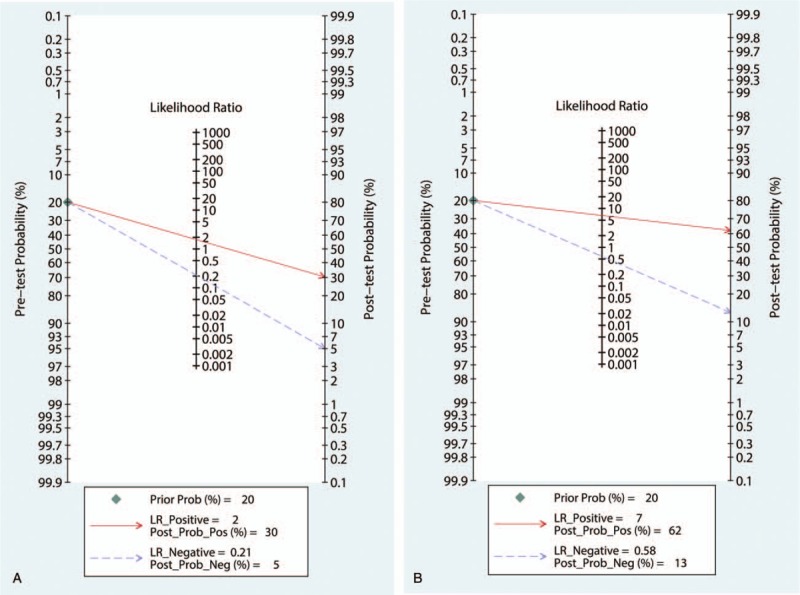
Fagan nomogram for urinary TIMP-2 and IGFBP7 test, showing post-test probabilities for AKI in critically ill patients. (A) urinary TIMP-2 and IGFBP7 = 0.3 (ng/mL)^2^/1000 and (B) urinary TIMP-2 and IGFBP7 = 2.0 (ng/mL)^2^/1000. IGFBP7 = insulin-like growth factor binding protein 7, TIMP-2 = tissue inhibitor of metalloproteinase 2.

**Figure 7:**
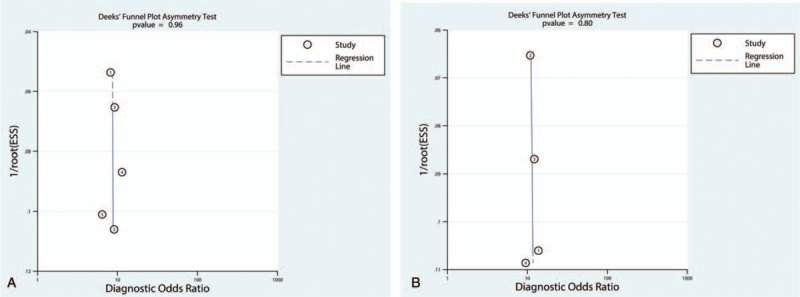
Funnel plot of publication bias.

When the pre-test probability of 20% was combined with the PLR and NLR, the post-test probabilities of urinary TIMP-2 and IGFBP7 ≥2.0 (ng/mL)^2^/1000 and <2.0 (ng/mL)^2^/1000 for AKI were 62% and 13%, respectively (Fig. 6B). Deeks’ test (Fig. 7B) showed a statistically non-significant value (*P* = .80), indicating that there was no potential publication bias.

### 3.6 Diagnostic accuracy of urinary TIMP-2 and IGFBP7 at cut-offs of 0.4 and 0.8 (ng/mL)^2^/1000

Two studies, with a total of 330 critically ill patients, assessed the diagnostic accuracy for AKI of a urinary TIMP-2 and IGFBP7 cut-off of 0.4 (ng/mL)^2^/1000. Because Meta-DiSc Stata software requires a minimum of 3 studies to compute SROC, the random effects model in Meta-DiSc was used to summarize estimates in this sub-meta-analysis. Pooling these studies, we found a sensitivity of 0.82 (95% CI 0.72–0.89), a specificity of 0.51 (95% CI 0.45–0.58), a PLR of 2.02 (95% CI 1.25–3.26), an NLR of 0.28 (95% CI 0.11–0.67) and a DOR of 8.05 (95% CI 3.94–16.46; *I*^*2*^ = 0) (Table 3).

**Table 3:**
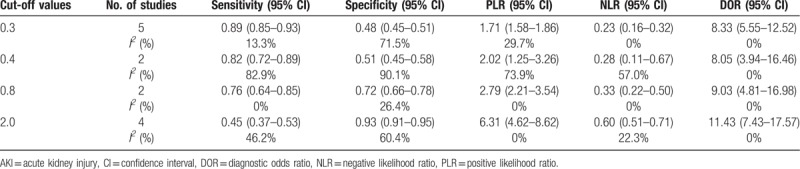
Diagnostic accuracies of urinary [TIMP-2] x [IGFBP7] for AKI at different cut-off values.

Two studies, with a total of 330 critically ill patients, assessed the diagnostic accuracy for AKI of a urinary TIMP-2 and IGFBP7 cut-off of 0.8 (ng/mL)^2^/1000. Pooling these studies resulted in a sensitivity of 0.76 (95% CI 0.64–0.85), a specificity of 0.72 (95% CI 0.66–0.78), a PLR of 2.79 (95% CI 2.44–5.00), an NLR of 0.33 (95% CI), and a DOR of 9.03 (95% CI 7.31–30.32, *I*^2^ = 0) (Table 3).

### 3.7 Sensitivity analysis

Sensitivity analysis was conducted in terms of definition of AKI, blind method, AKI threshold, time of measurement. The results were similar to the original, producing no obvious changes, when we excluded the definition of AKI (AKIN), blind method(no), AKI stage 1 or time of measurement (within 24 hours), respectively. In addition, the results showed overall diagnostic performance of urinary TIMP-2 and IGFBP7 at a cut-off of 2.0 (ng/mL)^2^/1000 is superior to urinary TIMP-2 and IGFBP7 at a cut-off of 0.3 (ng/mL)^2^/1000 (Table 4).

**Table 4:**
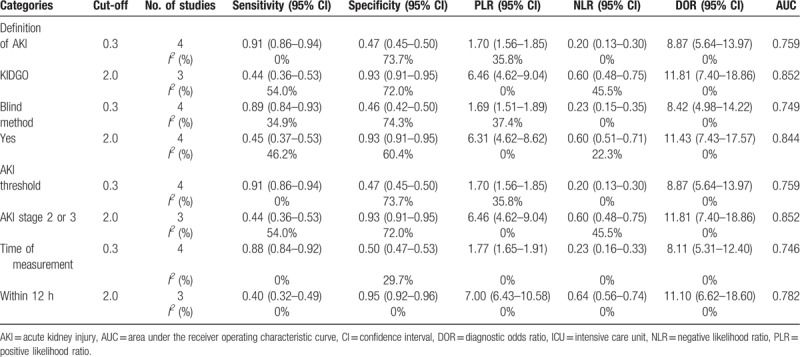
Results of sensitivity based on different criterion.

## 4 Discussion

The results of this meta-analysis confirmed the correlation of urinary TIMP-2 and IGFBP7 with AKI in critically ill patients, with a cut-off level of 2.0 (ng/mL)^2^/1000 being associated with optimal diagnostic accuracy for AKI. Generally, higher cut-off levels were associated with reduced sensitivity and increased specificity.

It is a general practice to determine the optimal cut-off value for a continuous predictive marker. Two cut-off values for urinary TIMP-2 and IGFBP7 are widely used in the diagnosis of AKI, 0.3 and 2.0 (ng/mL)^2^/1000. Urinary TIMP-2 and IGFBP7 > 0.3 (ng/mL)^2^/1000 measured within 24 hours of ICU admission was found to have a sensitivity of 95% for moderate or severe AKI, but its specificity for AKI was only 37.5% ^[16]^. False-positive effects are therefore frequently observed if this test is used inappropriately in low-risk patients. This cut-off for urinary TIMP-2 and IGFBP7 value would have greater predictive value in patients at high risk than at low risk for AKI. Urinary TIMP-2 and IGFBP7 at a cut-off of > 2.0 (ng/mL)^2^/1000 had a specificity of 86.1%, but a sensitivity of only 38%. Hence, the optimal cut-offs of urinary TIMP-2 and IGFBP7 levels for diagnosing AKI remain unclear.

Use of different cut-offs may allow urinary TIMP-2 and IGFBP7 concentrations to play several roles in clinical practice. A cut-off of 0.3 (ng/mL)^2^/1000 had good sensitivity (0.89), making it reliable in ruling out AKI, whereas a cut-off of 2.0 (ng/mL)^2^/1000 had good specificity (0.93), enabling it to confirm AKI. Furthermore, cut-offs of 0.4 and 0.8 (ng/mL)^2^/1000 showed relatively balanced sensitivity (0.82 and 0.76, respectively) and specificity (0.51 and 0.72, respectively). An overall evaluation showed that the cut-off of 2.0 (ng/mL)^2^/1000 had the highest DOR (11.43; 95% CI 7.43–17.52) when compared with other cut-off thresholds and had a higher AUC (0.844) than the cut-off of 0.3 (ng/mL)^2^/1000. A comparison of the diagnostic accuracy of the 4 cut-off values showed that 2.0 (ng/mL)^2^/1000 was optimal, as it had the highest DOR and AUC.

Fagan nomogram showed that likelihood ratio and post-test probability were both low for cut-offs of 0.3 (ng/mL)^2^/1000. A PLR of 2 indicates that a person with disease is twice as likely to have a positive test result as a healthy person. At a pretest probability of 20%, the post-test probability for a positive test result was 30%. Likewise an NLR of 0.21 reduces the post-test probability to 5% for a negative test result. In contrast, both the likelihood ratio and post-test probability were high at 2.0 (ng/mL)^2^/1000. A PLR of 7 indicates that a person with disease is 7 times more likely to have a positive test result as a healthy person. At a pretest probability of 20%, the post-test probability for a positive test result was 62%. Likewise, an NLR of 0.58 reduces the post-test probability to 13% for a negative test result. Thus, a urinary TIMP-2 and IGFBP7 cut-off of 2.0 (ng/mL)^2^/1000 had the highest overall diagnostic accuracy.

Several recent meta-analyses^[23–29]^ have shown that urinary TIMP-2 and IGFBP7 are useful in the early diagnosis of AKI. Those studies, however, included a variety of clinical settings (e.g., cardiac surgery, critically ill patients and emergency department), age groups (i.e., children vs adults) and cut-offs ranging from 0.3 to 2.0 (ng/mL)^2^/1000). Our analysis differed, in that it focused on a single clinical setting (critically ill patients only) and age group (adults).

Strengths of this study included the extensive and systematic literature search, the determination of the overall quality of the original studies and the finding that most of the included studies had a low risk of bias and applicability concerns. Second, this meta-analysis included one additional trial that was published recently and therefore not included in previous meta-analyses. As the latest and most comprehensively updated meta-analysis, the present study reinforces the results of previous meta-analyses. Third, we registered the protocol of this study on PROSPERO. A registered protocol may increase the transparency and quality of meta-analysis. Fourth, the present meta-analysis had lower heterogeneity than previous meta-analyses, based on the same cut-off points, and with similar sensitivity and specificity. The probable discrepancy may be due to the pooling by previous meta-analyses of different optimal cut-off values. Finally, we found that a urinary TIMP-2 and IGFBP7 cut-off point of 2.0 (ng/mL)^2^/1000 showed better overall performance than other cut-off points.

This study also had several limitations. First, a limited number of articles were included. The combination of urinary TIMP-2 and IGFBP7 is a relatively novel marker for the detection of AKI. Only 2 studies were included in the sub-meta-analysis that evaluated the ability of urinary TIMP-2 and IGFBP7 cut-off points of 0.4 and 0.8 (ng/ml)^2^/1000 to predict AKI in critically ill patients, which may have reduced the credibility of this analysis. Multicenter studies including large numbers of patients are required to confirm our findings. Second, the sampling times in the included studies differed, and we did not explore the optimal time to measure urinary TIMP-2 and IGFBP7 in the diagnosis of AKI in the same population and at the same cut-off point. Finally, although cut-offs of 0.3 (ng/ml)^2^/1000 and 2.0 (ng/ml)^2^/1000 were most commonly reported, other cut-off values may show better diagnostic accuracy.

## 5 Conclusion

Urinary TIMP-2 and IGFBP7 are valuable biomarkers for the early diagnosis of AKI. A urinary TIMP-2 and IGFBP7 cut-off point of 2.0 (ng/mL)^2^/1000 may show optimal overall performance because of high DOR and AUC. Nonetheless, further studies are required to determine the optimal cut-off of urinary TIMP-2 and IGFBP7 for the diagnosis of AKI. Multicenter clinical trials with larger sample sizes and high-quality evidence are needed.

## Author contributions

Study concept and design: QHW, DQZ and YY. Acquisition of data: QHW, DQZ, YY and LFG. Analysis and interpretation of data: QHW, DQZ and YY. Drafting of the manuscript: QHW, DQZ and LFG. Critical revision of the manuscript for important intellectual content: QHW and YY. Statistical analysis: QHW, DQZ and YY. Administrative, technical, and material support: QHW, DQZ and LFG. Study supervision: QHW. All authors have read and approved the manuscript for publication.

**Conceptualization:** Dongquan Zhang, Quanhong Wang.

**Data curation:** Dongquan Zhang, Quanhong Wang.

**Formal analysis:** Dongquan Zhang, Quanhong Wang.

**Funding acquisition:** Dongquan Zhang, Quanhong Wang.

**Investigation:** Dongquan Zhang, Quanhong Wang.

**Methodology:** Yuan Yuan, Quanhong Wang.

**Project administration:** Yuan Yuan, Quanhong Wang.

**Resources:** Yuan Yuan, Quanhong Wang.

**Software:** Yuan Yuan, Quanhong Wang.

**Supervision:** Yuan Yuan, Quanhong Wang.

**Validation:** Yuan Yuan, Longfei Guo, Quanhong Wang.

**Visualization:** Longfei Guo, Quanhong Wang.

**Writing – original draft:** Longfei Guo, Quanhong Wang.

**Writing – review & editing:** Longfei Guo, Quanhong Wang.
